# Empathy mapping and hybrid persona creation: Understanding older adults' needs in technology design for ageing in place

**DOI:** 10.1016/j.csbj.2025.11.044

**Published:** 2025-11-20

**Authors:** Andrea Kastl

**Affiliations:** a Rosenheim Technical University of Applied Sciences, Germany; b University of Innsbruck, Austria

**Keywords:** Persona, Ageing in place, Empathy mapping, Older adults, Digital divide, Smart medical devices, AI

## Abstract

**Background:**

Technology yields the potential to support independence in later life and facilitate ageing in place. Barriers such as limited usability, varying levels of digital literacy, and persistent age-related stereotypes are documented to hinder technology adoption among older adults. User-centered design approaches, such as the persona method, offer strategies to address these challenges.

**Objective:**

Personas were created to inform the development of technologies supporting ageing in place by enhancing stakeholders' understanding of needs and technology experience among older adults.

**Methods:**

The personas were developed through an iterative process, with this paper detailing the last stage, which employed the a PATHY technique as conceptual framework for content analysis. Eleven focus group discussions were conducted in southern Germany, of which nine were included in the analysis. These involved older adults in need of support, with orthopedic and neurological conditions, and in informal caregiving roles.

**Results:**

Five hybrid personas were created to represent the target groups' experiences related to ageing in place, each reflecting needs, problems, existing solutions, habits, sociodemographic characteristics, and dimensions of technology experience. Key factors influencing technology experience and uptake among the personas were identified, including the social context and availability of support, the perceived trade-off between safety and digital privacy, and the persistence of the digital divide.

**Conclusion:**

The findings underscore the necessity of adaptable, context-sensitive technology solutions, displayed by the personas’ needs, pains, routines and technology experiences. Thereby, the hybrid personas reinforce the importance of a more relational and context-aware approach in user-centered design.

## Graphical Abstract

**Figure:**
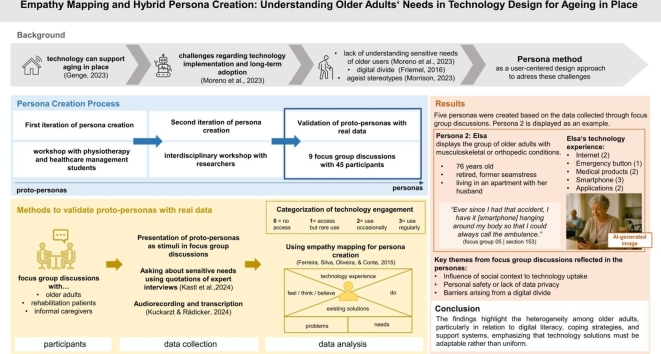

## 1 Introduction

Ageing in place (AIP), commonly defined as the ability to live safely, independently, and comfortably in one’s own home or community [1], has become a widely endorsed goal in ageing policies. It is increasingly recognized as a response to the healthcare demands of an ageing population, particularly in terms of delaying entry into long-term care facilities [2]. Across countries, a majority of older adults express a strong preference to remain in their own homes and familiar environments for as long as possible [2], [3], [4].

The definition of AIP has been critically examined in academic discourse, particularly for its underlying assumption that remaining in one’s own home is the most desirable option for AIP [5]. To address this limitation, Rogers et al. [5] advocate for a more inclusive definition: *'one’s journey to maintain independence in one’s place of residence, as well as participate in one’s community'*. This broader definition shifts the focus beyond the traditional emphasis on the physical location of “home” towards a more holistic understanding of ageing in place that encompasses maintaining autonomy and active participation within the social environment. However, the realization of AIP depends on a range of factors that can act as both enablers and barriers [6]. These include accessibility of dwelling, safety, availability of social support and networks, primary healthcare access and adequate funding [7].

In response to these multifaceted needs, a variety of technologies have emerged as promising tools to support AIP, such as wearable devices, assistive robotics, sensor-based systems integrated into Internet of Things (IoT) environments, telemedicine (platforms), information and communication technologies (ICT), and assistive devices (including high-tech and low-tech tools) [8], [9], [10]. These technologies can contribute positively to the maintenance of independence, improve health-related quality of life among older adults [11] and support informal caregivers by enhancing safety, communication, and health monitoring [12].

Despite a growing body of evidence supporting the potential of technology to enable AIP, the adoption and sustained use of digital tools among older adults remains biased towards certain groups of devices. Most older individuals continue to rely on "low-tech" technologies such as landline telephones and television [13]. Although technology uptake in this population has increased since the onset of the COVID-19 pandemic [14], challenges to long-term adoption persist. These include access, low perceived usefulness, physical barriers, and the extent to which technologies meet older users’ specific needs [12], [14], [15]. Additional barriers to realizing the potential benefits of technology for AIP include varying levels of digital literacy [9], defined as the ability to use and operate digital tools [16], as well as unequal access to digital infrastructure and devices [17]. These factors contribute to the persistence of a phenomenon known as the digital divide [18], [19]. The digital divide refers to disparities in the access and use of information and communication technologies (ICT) among people or nations due to determinants such as age, income, gender, place of residence, and education [19], [20]. It is distinguished at three levels: (1) access to the internet and technologies; (2) digital skills and usage patterns; and (3) the ability to derive beneficial outcomes from using ICT [20]. These divides remain critical challenges in the equitable adoption of digital technologies among older adults. Factors identified as facilitators of technology use among older adults include technological knowledge, availability of social support, accessibility and socially driven motivation to engage with digital tools [14]. Moreno et al. recommend prioritizing privacy, usability, and addressing unmet needs in the development of technology for AIP, while avoiding replacing familiar technologies with which older adults are already comfortable using [12].

Although older adults who wish to age in place represent a highly diverse population, they are often conceptualized as a homogeneous group in both research and applied settings [19]. This generalization can result in a technology design that does not adequately address individual differences or user preferences. Additionally, ageist assumptions and stereotypes concerning older adults' ability or willingness to engage with technology may hinder inclusive and effective design [21]. Technologies that reinforce stereotypes are often met with resistance, as they conflict with older adults’ self-perception [15]. Collectively, these insights point to the need for more inclusive, user-centered design (UCD) approaches that recognize the heterogeneity of older adults and address not only functional needs but also contextual and infrastructural barriers to digital participation.

UCD is a design philosophy guided by a deep understanding of users’ needs and environments, as well as involving them throughout an iterative design lifecycle [22]. Already in 1985, three principles of design were recommended by Gould and Lewis: (1) early focus on users and tasks, (2) empirical measurement, (3) iterative design [23]. The persona method, as a UCD tool for communicating and representing users’ needs [24], is increasingly used in the development of health technologies for user involvement [21], [25]. The persona method originates from the fields of design, marketing, and human-computer interaction (HCI) [26], [27], [28]. Personas serve as representations of user types and are applied to help bridge communication gaps between stakeholders, including developers and designers [27].

Despite its widespread use, the persona method is primarily employed for design purposes rather than for research itself [29], [30], [31]. This study applies the persona method in qualitative research (see Fig. 2) and presents resulting hybrid personas to inform both future research and UCD. The persona method is coupled with empathy mapping to create personas of older adults in need of support, with orthopedic and neurological conditions, as well as older adults in informal caregiving roles [32]. Five personas were derived from qualitative data collected through focus group discussions. In line with a constructivist approach, the personas were created with the aim of facilitating understanding through feedback iterations and contextual adaptation [33]. They serve as both a medium of communication and an outcome of the research process.

The personas are intended to enhance stakeholders’ understanding of the diverse needs and levels of technology experience among older adults in need of support, with orthopedic and neurological conditions, as well as older adults in informal caregiving roles, and to promote empathy and more inclusive implementation practices. This focus does not imply a generalized representation of older adults; rather, it reflects an effort to address the needs of those whose experiences are often marginalized in technology design research and shaped by ageist assumptions in innovation discourses [34].

The primary objective is to inform the design and development of technologies that support AIP, and specifically within this study, to guide the equipment of residential competence centers with technologies.

## 2 Revisiting the persona method: methodological strengths and limitations

Personas are defined as fictional archetypes representing clusters of real users who share similar needs and characteristics [35]. Personas are therefore designed to represent segments within a target population. Their primary purpose is to facilitate a deeper understanding of prospective users, enabling designers, developers, and researchers to create user-centered solutions. Therefore, personas include not only demographics, but "put a face on the user" by illustrating goals, routines, and characteristics [35] and thereby connecting stakeholders with the target population [24]. Personas are usually conveyed using visual representations, positioning them as an innovative way to communicate research outcomes [33].

Recent studies create personas using qualitative, quantitative and mixed-methods approaches or artificial intelligence (AI) to generate personas [36], [37]. While Kirchem and Waack describe the creation of personas from a marketing and sales perspective in interdisciplinary teams using templates and rather qualitative data [38], other studies report on big data-driven [39], [40], or co-design approaches [27], [41]. This study applies a qualitative-hybrid approach to persona creation as quantitative approaches to persona creation have been critiqued for their limitations in conveying rich, narrative-driven insights and an understanding of motives. These insights are essential for fostering empathy among designers and developers, which is a key objective of persona development processes [30]. While the persona’s textual descriptions are human-created, the accompanying images are AI-generated in this study.

Previous research has suggested that personas can be used for strategic business development purposes within organizations, as well as for design, to include marginalized groups, and to encourage creative discussion [31], [42]. Overall, the use of personas in research and product design offers benefits such as building empathy and (emotional) understanding among research or design teams for the target audience [24], [26], [43]. Personas help in communication and decision-making in UCD processes about what features address the users' needs best, and which features can be discarded, thereby contributing to greater efficiency and better design decisions [24], [27], [44]. Therefore, personas can be an important tool for UCD, as well as for research communication, especially when marginalized user groups are difficult to engage with or involve [27], [42], [45].

## 3 Materials and methods

**Fig. 1:**
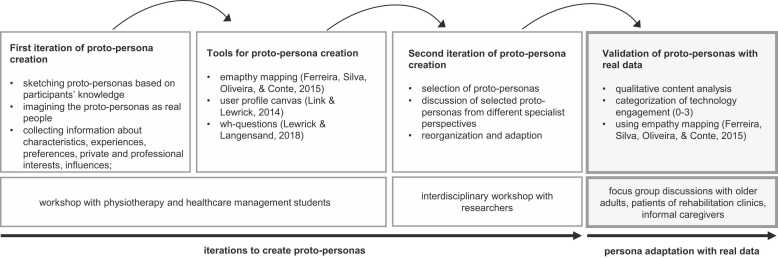
Process model proto-persona creation and validation (author's presentation).

The methods section outlines the iterative development of personas through empathy mapping. Proto-personas generated during steps 1–3 (see Fig. 1), which are not yet based on empirical data, served as stimuli for focus group discussions in step 4. The qualitative data obtained from these discussions informed the subsequent adaptation of the initial proto-personas, resulting in the personas presented in the results section.

The persona method reported here was part of the DeinHaus 4.0 – Oberbayern (YourHome 4.0 – Upper Bavaria) research project. There, personas provided information about the research project’s target groups: older adults, patients of rehabilitation clinics, and people with disabilities, to ensure that model homes’ equipment aligned with the needs of the intended users. Although informal caregivers were not a primary target group, a persona was developed due to their key role in home care [46]. The approach was approved by the Joint Ethics Committee of the Bavarian Universities of Applied Sciences (GEHBa-202104-V-022).

### 3.1 Iterations of persona creation

The first step of the persona development process involved a workshop with physiotherapy and healthcare management students as part of an online lecture at the Rosenheim University of Applied Sciences in November 2020. The students were in advanced stages of their bachelor’s program and therefore had exposure to healthcare practice. Their role was to create the proto-personas of older adults and informal caregivers as an initial, exploratory step in the design process. The 3.5-hour workshop was conducted online, as face-to-face meetings in larger groups were restricted due to COVID-19. After an introduction to the persona method and the aim of the research project DeinHaus 4.0 – Oberbayern and its target groups, students were asked to create a *user persona*, e.g. representing an older adult with mobility limitations and a network persona, e.g. representing an informal caregiver. In the workshop, the students worked in three groups, with each group consisting of four students. During the workshops, several templates and tools such as a *user profile canvas* [47]*, a persona empathy map* [48] or *wh-questions* [49] were introduced to the students as a guideline for the development and structured presentation of the proto-personas [33]. The students were instructed to use at least two of the introduced templates. As a result, the students developed three user and three network proto-personas.

In a second iteration workshop, the students' proto-personas were reused and aligned with the target groups of the research project DeinHaus 4.0 – Oberbayern. During the workshop with six members of the research project's interdisciplinary team, consisting of researchers with backgrounds in nursing, physiotherapy, mechatronics and psychology, five proto-personas were developed, critically discussed, and adapted to the feedback (see Fig. 1).

Fig. 1 depicts the overall persona creation process, including the first two iterations, but this manuscript primarily focuses on the last step of persona creation, where empirical data serve as the foundation. Based on the focus group discussions with older adults, patients of rehabilitation clinics, and informal caregivers, the proto-personas were adapted using the PATHY technique, which combines empathy maps and personas [32], [48].

### 3.2 Applying the proto-personas in qualitative research

**Fig. 2:**
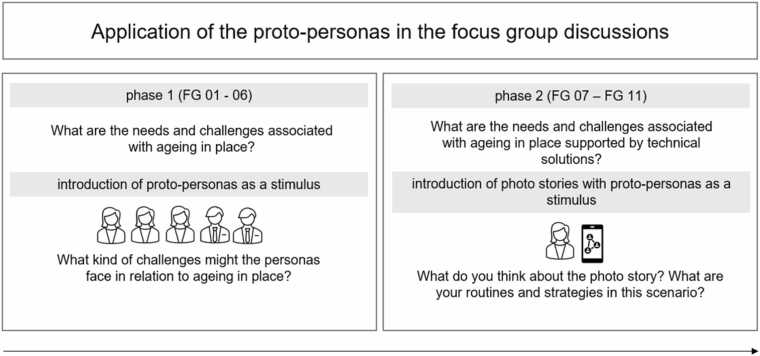
Proto-personas as a stimulus in focus group discussions (author’s presentation).

Proto-personas were employed as stimuli in focus group discussions with the project’s target groups (Fig. 2). To this end, eleven focus group discussions were held in homogeneous group constellations, with a group size of between four and seven participants, and with different participants in each group, from August 2021 to January 2023. Within the *first phase* (FG 01 – FG 06), the discussions were designed to explore and understand the needs and challenges associated with independent living and AIP. At the outset of each session, the proto-personas were introduced and presented on A4-sized sheets to create a comfortable atmosphere conducive to open dialogue. Participants were then asked to reflect on the potential difficulties the proto-personas might face in relation to AIP. By focusing initially on the perceived needs of others rather than their own, participants were encouraged to open up more freely. This approach draws on the concept of "othering" [50] and, in this context, was employed as a strategy to facilitate discussion around personal and sensitive experiences [51].

After the initial discussion following the presentation of the proto-personas, participants could choose from a pool of additional discussion topics. The pool of topics was developed based on insights from a previous interview study with key stakeholders in AIP, including outpatient and inpatient nurses and informal caregivers [52].

The *second phase* of focus group discussions (FG 07–FG 011) was conducted to explore the needs of older adults in the context of ageing in place supported by technological solutions. To facilitate this, proto-personas were introduced using photo stories that depicted everyday scenarios in which technology might offer support. Participants were invited to reflect on their own routines and technology use in relation to the situations illustrated in the photo stories. Each focus group engaged with three to six distinct photo stories.

### 3.3 Data generation

Focus group discussions were conducted to explore the needs and challenges faced by older individuals who wish to age in place and by older adults in informal caregiving roles. Prior to the focus group sessions, potential participants received detailed information about the research project, the objectives of the discussions, and the data protection procedures. Informed consent was obtained from all participants. Focus group participants were recruited through organizational and medical staff at rehabilitation clinics, nursing homes, and through self-help groups in Bavaria. The staff was informed about the research project. The local administration office, a cooperating partner of the research project, supported the recruitment process.

The discussions took place in rooms with a round table, ensuring that participants and the two moderators were positioned at an equal level for effective communication and interaction. Each focus group discussion lasted between one and a half and two hours and was audio-recorded. At the beginning of each focus group discussion, participants were asked to provide information about their gender, age, and place of residence (urban or rural) on a poster.

While the overall research design and data collection were predefined by the project scope, thematic saturation was assessed during data analysis. After the first several groups, no new categories relevant to the research questions (Fig. 2) emerged, indicating data category saturation within the available material. This observation is consistent with a study on data saturation in focus groups, which showed that 81–90 % of all codes were identified within the first three focus groups [53].

### 3.4 Participant characteristics

Data collection comprised nine focus group discussions with a total of 45 participants. Table 1 shows the central recruitment characteristics that guided the purposive formation of the focus groups.

**Table 1:** Constellations of focus group discussions.

**Recruitment characteristics**	**n, Number of participants**	**Focus group discussions**
Seniors living in a nursing home	4	FG 01
Patients at a neurological rehabilitation clinic	22	FG 02, FG 08, FG 09, FG 10
Patients at an orthopedic rehabilitation clinic	9	FG 05, FG 06
Seniors living independently at home	5	FG 03
Informal caregivers	5	FG 11

Table 2 reports the participants’ sociodemographic characteristics. The participants ranged in age from 50 to 59–80–89 years. Twenty-four participants identified as female and twenty-one as male; no participant identified as diverse. Seventeen participants reported residing in rural areas, while twenty-eight reported to live in urban settings.

**Table 2:** Participant sociodemographic characteristics.

**Age**	**Female**	**Male**	**Urban Area**	**Rural Area**	**Total Number**
50–59	5	3	5	3	**8**
60–69	10	7	9	8	**17**
70–79	4	9	9	4	**13**
80–89	5	2	5	2	**7**
**Total Number**	**24**	**21**	**28**	**17**	**45**

### 3.5 Data analysis

The recorded data were transcribed according to the transcription rules of Kuckartz [54]. The transcripts of nine focus group discussions with a total of 45 participants were used to adapt the proto-personas with real data.^1^ To analyze participants' discussions, a deductive-inductive content analysis was applied utilizing the software MAXQDA [55]. The PATHY technique provided the initial, deductive coding framework: *needs, problems, technology experience, do, think, feel and existing solutions* [32]. In a second round of inductive coding, categories were refined and additional subcategories were captured. For data protection reasons, *sociodemographic information* was not derived from the transcripts but was generated by the author.

• *Sociodemographic data:* A detailed description of the persona expressing an ageing in place desire, including demographic data, alongside physical or mental health conditions.
• *Feel & Think:* What does the persona think and feel? What are worries, dreams, fears?
• *Do*: What is the persona's everyday life like? What is their environment like, and what are their routines and hobbies?
• *Problems*: What barriers and problems does the persona face, especially related to ageing in place?
• *Needs:* What are the persona’s needs? What does the persona need to overcome problems/ barriers to ageing in place?
• *Existing solutions:* Are there solutions that help the persona to solve these problems?
• *Technology experience*: An overview of the technologies currently utilized by the persona.

The individual fields were then refined inductively. To display older adults’ technology experience in persona creation, the author developed four categories (0−3) to describe each persona’s orientation toward technology. The categorization represents an adaptation of the four-profile model of technology engagement originally proposed by Serino et al. [56], contextualized for AIP by describing frequency of use [13]. The categories’ definitions were informed by a combination of existing literature [13] and empirical patterns observed in the focus group discussions. In the context of the digital divide, evidence indicates that older adults may lack both awareness of and access to technologies that facilitate AIP [19]. Consequently, category 0 was defined to capture this condition, representing the absence of access to relevant technological resources (Table 3). The different categories of technologies were informed by the technologies mentioned by the participants in the focus group discussions (see Table 5). The category of technology engagement assigned to each persona was derived from the predominant patterns of technology experience that emerged during the respective discussions.

**Table 3:** Categorization of technology engagement (author's presentation).

**Categories**	**Description of technology engagement**
0	No access to relevant technology
1	Has access but does not use it, or uses it rarely (less than once a week)
2	Uses technology occasionally (e.g., once a week or for specific tasks)
3	Uses technology regularly (e.g., daily or multiple times per week)

The adaptation of proto-personas was conducted by selecting one to three focus group discussions per persona, based on the alignment between participant characteristics and the attributes represented in the respective persona (see Table 4). Each aspect of the proto-persona was then compared with the coding results, and adapted to the real data. To better represent the focus group participants’ gender ratio, the gender of persona 5 was changed from female to male with a corresponding name update.

**Table 4:** Persona creation through qualitative data collected through focus group discussions (author’s presentation).

**Proto-Personas used in the research project DH 4.0**	**Target group the persona is representing**	**Qualitative data chosen for proto-persona adaptation**	**Final Personas**
Persona 1 Hermann Huber	Older adults residing in long-term care facilities	FG 01, 03	Persona 1 Hermann Huber
Persona 2 Elsa Müller	Older adults with musculoskeletal or orthopedic conditions	FG 05, 06	Persona 2 Elsa Müller
Persona 3 Annegret Maier	Older adults with neurological disorders	FG 02	Persona 3 Annegret Maier
Persona 4 Maria Weber	Older adults in informal caregiving roles	FG 11, 03	Persona 4 Maria Weber
Persona 5 Moni Oberbauer	Older adults with neurological disorders	FG 08, 09, 10	Persona 5 Thomas Fischer

### 3.6 Generation of persona images using AI

The last step of the persona creation was the addition of persona images to the textual component. To generate the persona images, a large language model was used (ChatGPT LLM 4.0). Five aligning prompt structures, but tailored to the specific characteristics of each persona, were developed and applied.

### 3.7 Research team and reflexivity

The author (AK, f) holds a background in physiotherapy and international health and social management and facilitated all discussions as a moderator. Team colleagues from the DeinHaus 4.0 - Oberbayern research project provided support as second moderators. In this single-authored study, all coding procedures were carried out solely by the author.

## 4 Results

**Fig. 3:**
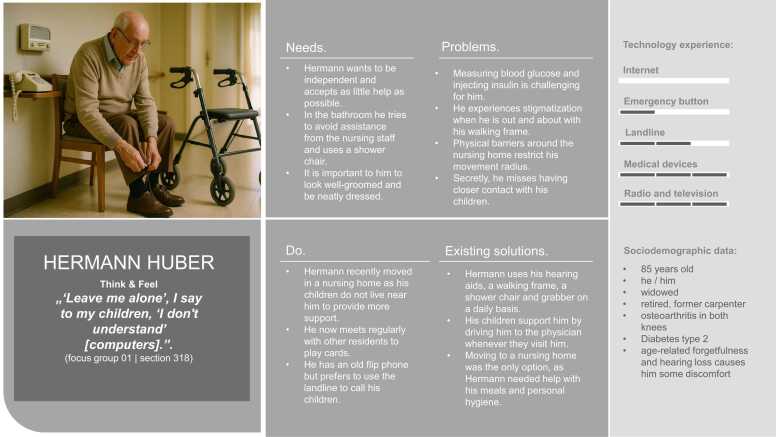
Persona Hermann Huber (authors’ presentation; image AI generated).

**Fig. 4:**
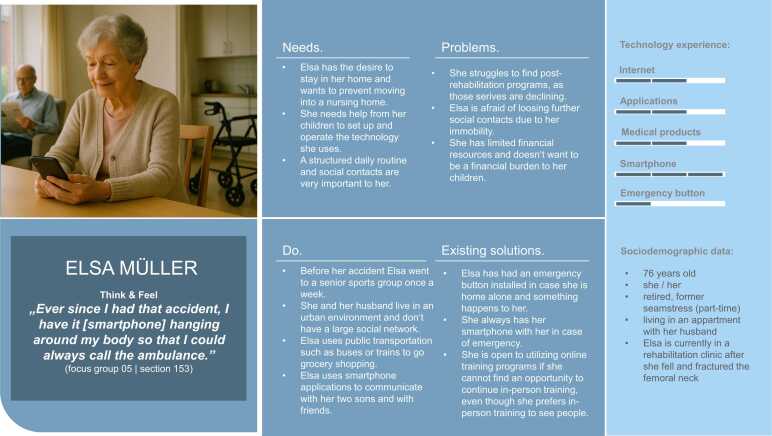
Persona Elsa Müller (author’s presentation; image AI generated).

**Fig. 5:**
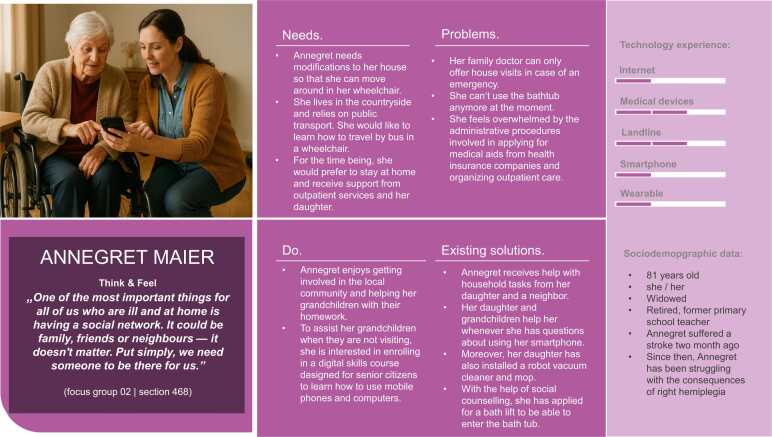
Persona Annegret Maier (author's presentation; image AI generated).

**Fig. 6:**
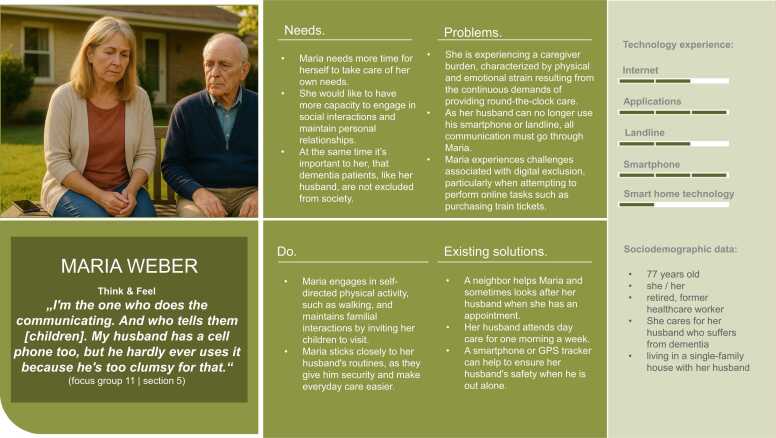
Persona Maria Weber (author's presentation; image AI generated).

**Fig. 7:**
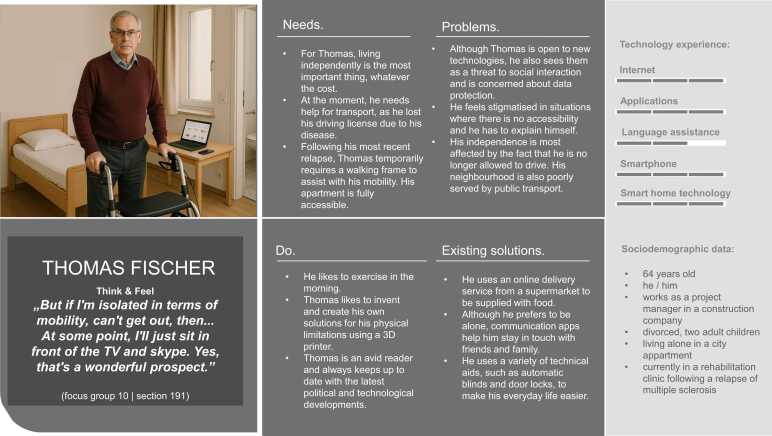
Persona Thomas Fischer (author's presentation, image AI generated).

Overall, five hybrid personas were developed to represent the research project’s target groups. The text parts of the personas were created based on the data collected through the focus group discussions. Fig. 3, Fig. 4, Fig. 5, Fig. 6, Fig. 7 present the personas Hermann Huber, Elsa Müller, Annegret Maier, Maria Weber, and Thomas Fischer, combining the human-created text and AI-generated images to hybrid personas. During the inductive coding process, three sub-themes to the category *technology experience* were identified: (1) social contexts as determinants of technology uptake, (2) tensions between safety benefits and data-privacy concerns, and (3) expressions of the digital divide. These sub-themes were incorporated into the persona descriptions.

The focus group discussions revealed several domains of technology application mentioned by participants. The technology-engagement categories were used to present the frequency of technology use for each persona.

Table 5 provides an overview of the technological products mentioned across the focus group discussions. The table summarizes whether a specific type of technology or service was brought up, indicated by a checkmark (✓). The categories serve as a foundation for the development of user personas and the presentation of their technology experience. This binary representation indicates only the presence or absence of references to specific technologies within the focus group discussions. For each persona, the five most relevant technology categories were identified and assessed according to the technology-engagement categories.

**Table 5:** Technology experience among older adults: emergent themes in focus group discussions (author’s presentation).

	**Persona 1** Hermann Huber (FG 01, 03)	**Persona 2** Elsa Müller (FG 05, 06)	**Persona 3** Annegret Maier (FG 02)	**Persona 4** Maria Weber (FG 11, 03)	**Persona 5** Thomas Fischer (FG 08, 09, 10)
Smartphone use	✓	✓	✓	✓	✓
Use of medical products	✓	✓	✓	✓	
Tablet/ computer use	✓	✓		✓	✓
Internet access and use	✓	✓		✓	✓
Application use	✓	✓		✓	✓
Landline telephone use	✓			✓	
Television use	✓				✓
Emergency button use	✓	✓	✓	✓	✓
Use of online services		✓			
Use of wearables			✓		✓
Use of smart home technology			✓	✓	✓
Use of artificial intelligence					✓

## 5 Discussion

This study aimed to develop personas representing needs, challenges, and technology experiences of older adults in need of support, including older adults with orthopedic or neurological conditions, as well as older adults in informal caregiving roles, to inform the UCD of technology to support AIP. Accordingly, the persona method is discussed as a qualitative research tool for synthesizing and communicating user experiences and perspectives.

### 5.1 Reflecting on stereotypes and stigmatization in persona-driven design

Personas can serve as powerful instruments to stimulate participation and facilitate creative discussions in research with older adults [27], [42], [44]. However, they also bear the risk of reinforcing limiting representations such as gender or age-related stereotypes [57], [58], [59], [60] and reconstructing internal political realities rather than real user needs [61] if not critically developed and reflected upon.

The personas created in this study aim to reflect the diversity of older adults in need of support, including individuals with neurological and orthopedic conditions and those in caregiving roles, in terms of their needs, living situations, preferences, and technology experience.

While personas are valuable tools in user-centered design for representing user needs throughout product design [35], they are by definition archetypal and not intended to represent the full complexity of individual experiences within a given user group. Even within a well-defined target population, it may be that no real person will identify with a given persona [62]. In this context, the aim was not to represent the entire older adult population or all informal caregivers, both of which are heterogeneous groups [63], but to develop representative user proxies that can inform design processes for aging in place technologies within specific subgroups. A previous study highlights how age-oriented technologies frequently reproduce images of active ageing and risk marginalizing older adults who deviate from those ideals, emphasizing the importance of intentionally incorporating voices of older people with health, mobility, or care needs [64].

When reflecting on potential reproduction of stereotypes, the application of AI-generated visuals to complement the human-created textual components of personas must be discussed critically. Prior research has demonstrated how visuals of older adults generated by AI reinforce digital ageism, for instance, by predominantly portraying negative emotional expressions and lacking diversity in representation [65]. In response to these concerns, the present study employed specifically developed prompts informed by the textual persona component to generate persona images that closely resemble focus group participants. Most of the resulting persona images show happy, content or neutral facial expressions. However, the image representing the informal caregiver persona “Maria” shows a worried expression. This visual outcome was intentional, as the prompt included her high level of burden and exhaustion, a recurring theme identified in the focus group discussion with the informal caregivers. Therefore, I argue that this representation displays one reality of caregiving, rather than reinforcing digital ageism [65]. In doing so, they challenge prevailing stereotypes and align with current research advocating for a more nuanced understanding of older populations [21].

### 5.2 Social contexts as determinants of technology uptake

The findings, interpreted through a social constructivist lens, indicate that older adults’ engagement with technology is a socially constructed process shaped by interpersonal relationships, particularly support from family members in setting up, explaining, and co-using digital devices. These insights align with earlier studies demonstrating that social networks can enhance technology uptake by providing encouragement, practical help, and shared norms around usage [13], [14], [66].

However, the social dimension also proved to be a potential barrier. For some, technology-mediated communication was seen as a threat to meaningful social interaction, or it placed an additional burden on informal caregivers, particularly when digital platforms had to be used on behalf of a spouse or parent. In response to limited digital competencies, some participants expressed openness to in-person training formats, including online options, if no in-person alternatives were available, highlighting a willingness to engage with technology when social support and relevance are perceived. These findings are consistent with Chan et al. [66], who recommend person-centered peer-to-peer technology training for older adults.

### 5.3 Safety-driven technology uptake and the dilemma of data privacy

Technology was frequently adopted for its perceived contribution to personal safety. Examples included the use of smartphones to stay connected, GPS trackers in dementia care, and smart home devices such as automatic door locks. These findings align with broader trends in the field of gerontechnology adaptation, where technologies that support safety, especially in emergencies or life-threatening situations, are likely to promote acceptance among older adults [67].

At the same time, concerns about digital privacy and data protection, such as fears around internet security, password management, and surveillance, tempered enthusiasm for these tools. Privacy-related concerns have been recognized in prior research as a significant barrier to the adoption of technology among older adults [66], [68]. This could also explain why technologies such as wearables and smart home technologies seem to be less adopted by focus groups participants, as depicted in Table 5. Persona Thomas expresses discomfort with data-sharing aspects, echoing existing literature that calls for transparent, user-centered data governance to build digital trust in ageing populations [69].

### 5.4 Between digital divide and (low-tech) technology uptake

The personas illustrate challenges voiced by the focus group participants such as lack of internet infrastructure in rural areas or limited connectivity in nursing homes, which can be assigned to the first-level digital divide [19], [20], [70]. In addition, difficulties related to the second-level digital divide [19] were also evident, including cognitive overload, when navigating online platforms for administrative tasks, such as managing finances or organizing transportation [20].

Table 5 provides an overview of technologies mentioned by the focus group participants. Many older adults described their engagement with low-tech solutions, ranging from medical products such as walkers and hearing aids to emergency buttons, landline telephones, and television, which are perceived as both familiar and reliable, aligning with earlier studies in the field of technology diffusion and adaptation [13]. Even though reported frequently, some participants perceived, in particular, the use of a walking frame as stigmatizing [15]. The regular use of smartphones for written communication (e.g., messaging, email) in parallel with landlines further highlights adaptive strategies that blend old and new technologies.

Wearables, smart home technologies, or artificial intelligence were scarcely mentioned by the participants in comparison to the emergency button or smartphone (see Table 5). These disparities in technology use may be attributed to the digital divide. Although many older adults already use devices such as smartphones, ongoing technological advancement contributes to the persistence of inequalities between “early adopters”, often younger people, and “laggards”, who are oftenolder adults [71].

However, as Moreno et al. [12] suggest, technologies that are perceived as replacing rather than complementing familiar tools may face resistance, even if they offer superior functionality. Additional insights emerged around technology adoption motivated by functional needs such as domestic hygiene (e.g., robot vacuum cleaners) and personal mobility (e.g., bath lifts, walking frames). In such cases, relatives often played a role not only in facilitating access but in actively installing and configuring devices.

### 5.5 Deployment of user personas in UCD

In this study, I suggested the combination of empathy mapping and personas as an element of UCD to inform communication and design choices. In line with UCD principles [22], personas are derived from focus group discussions involving older adults with orthopedic or neurological conditions or in caregiving roles, incorporating real user inputs. Context-specific findings and information about technology experience were captured in the personas’ attributes.

The personas developed can serve as a communication tool among interdisciplinary teams and help researchers and designers to connect with prospective users of AIP technologies, which was identified as a barrier to UCD processes by earlier studies [24]. Specifically, the personas provide insight into which technologies are already in use and familiar to older adults, giving designers an understanding of the technological functions with which this user population is comfortable. This is particularly important given Moreno et al.’s recommendation that new technologies should not replace existing ones but rather complement and build on those already accepted by users [12]. For example, Persona Hermann Huber uses radio and television on a daily basis and medical devices, such as hearing aids. He uses the landline telephone at least once a week; an emergency button is installed, but he doesn’t use it. He has no internet access. Moreover, applying the PATHY method to persona creation informs designers about Hermann’s problems, needs and routines, and identifies potential design spaces that are likely to be meaningful for older adults like Hermann and that new technologies should address.

The findings of this study align with recent discourses about UCD theory, suggesting a value-sensitive design approach that focuses on human values and what is meaningful to people in life [72]. An essay criticized ambient assisted living technologies for failing to address the main needs of potential users, such as social relationships, a sense of belonging, purpose in life, and privacy, in the context of human-computer interaction (HCI) [73]. By presenting personas that display social contexts and fundamental needs of older adults who require support, this study adds to UCD theory, alongside other studies, by calling for a more relational and contextual understanding of user-centeredness in design practice [41], [74].

In terms of policy recommendations, this study’s results point out the need for enhancement of digital infrastructure, especially in rural areas, as well as training and support programs embedded in social contexts of older adults, given the substantial influence of social factors on technology adoption and use [13], [66]. More broadly, the findings align with healthcare policies demanding patient-centered processes, including approaches that integrate patient-generated health data [75].

### 5.6 Limitations and future research

As this study adopts a social constructivist framework, it is important to reflect on how this epistemological stance intersects with the chosen methods. Focus group discussions are valuable for exploring participants’ opinions and tacit knowledge [76], but involve limitations that may be considered when interpreting the findings. Focus group participants might withhold dissenting views or sensitive topics due to social desirability bias [77] and the influence of group dynamics [78]. Consequently, it is important to acknowledge that the resulting personas may not fully capture the diversity or less socially acceptable perspectives within the target population.

Nevertheless, the persona method also presents practical limitations. The development process is resource-intensive and often tailored to the specific context of a single project, which may limit its transferability to other contexts [25]. Thus, a prior study recommends a careful and context-specific use of personas, noting their potential to engender political conflicts [79]. Existing literature has questioned the scientific rigor and validity of the persona method, particularly to determine how accurately a persona reflects the target population [40], [79]. To address this limitation, this study created personas using qualitative data, and documented each step from proto-personas to final personas to enhance methodological transparency. Further, concerns about the appropriate use of personas among design teams are raised [79]. An interview study with fourteen UCD practitioners showed that personas were mainly used for communication purposes outside UCD teams rather than for design, as personas are criticized for being too impersonal and abstract for design activities. However, these results did not apply to practitioners who had received persona training [80]. This study extends this discourse by demonstrating how personas can be integrated into qualitative research processes, for example, as stimuli in focus group discussions. Future studies could explore how empirically grounded personas are used in UCD and research settings.

The analytical process was conducted by a single author, which may introduce subjective bias. To mitigate this, the author performed the analytical coding twice, separated in time, to enhance reliability. Nevertheless, triangulation with additional researchers or participant validation could further strengthen the credibility of the findings.

Participants were recruited from various contexts, with five out of ten focus group discussions being conducted with patients of a neurological rehabilitation clinic. Consequently, two personas were developed for this group to reflect the diversity captured within it. The uneven recruitment process may have limited the representation of other user groups. Nevertheless, it was decided that the personas should reflect the empirical distribution of participants. As previously discussed, personas and their contextualization require critical reflection to avoid reinforcing stereotypes or oversimplifying user groups. Therefore, this study highlights the importance of developing a broader range of personas to better capture user heterogeneity, such as that found among informal caregivers [63].

## 6 Conclusion

This study contributes to UCD theory and practice by demonstrating how contextually grounded personas can serve as tools for considering fundamental needs, values, and experiences of older adults who require support in the development and design decisions for AIP technologies. The analysis identifies key factors influencing technology uptake among the personas, including the social context and availability of support, the perceived trade-off between safety and digital privacy, and the persistence of the digital divide. The findings reinforce the heterogeneity within the population of older adults in need of support in relation to digital literacy, coping strategies, and support systems, emphasizing that technology solutions must be adaptable rather than uniform. While personas offer valuable insights and foster empathy and communication among design teams regarding the target group in UCD, their development requires careful consideration to avoid reinforcing stereotypes and to ensure relevance beyond specific project contexts. Future research should focus on expanding persona diversity and on refining persona methodologies to enhance their reusability, thereby improving their potential to contribute to inclusive and context-aware UCD.

## CRediT authorship contribution statement

**Andrea Kastl:** Writing – review & editing, Writing – original draft, Visualization, Validation, Methodology, Investigation, Formal analysis, Data curation, Conceptualization.

## Declaration of Generative AI and AI-assisted technologies in the writing process

During the preparation of this work the authors used ChatGPT – Scholar AI in order to improve the readability and language of the manuscript. After using this tool, the authors reviewed and edited the content as needed and take full responsibility for the content of the published article.

The persona images presented in the results section were generated using AI (ChatGPT LLM 4.0), with each image created based on a consistent prompt structure tailored to the specific characteristics of each persona.

## Funding

This work was supported by the Bavarian State Ministry for Health, Care and Prevention [grant number: G64e-G8300–201868–14].

## Declaration

This work was conducted and written solely by a single author (Andrea Kastl), who is fully responsible for all aspects of the study and its content.

## Declaration of Competing Interest

The author declares no competing interests.

## Footnotes

1 Focus group 04 was excluded from the analysis due to audio recording failure, which resulted in insufficient data quality for transcription and analysis. Focus group 07 was excluded from this study, as the AIP framework does not sufficiently capture the distinct support needs and living contexts of individuals with intellectual disability.
